# Acute Administration of Non-Classical Estrogen Receptor Agonists Attenuates Ischemia-Induced Hippocampal Neuron Loss in Middle-Aged Female Rats

**DOI:** 10.1371/journal.pone.0008642

**Published:** 2010-01-08

**Authors:** Diane Lebesgue, Michael Traub, Maxine De Butte-Smith, Christopher Chen, R. Suzanne Zukin, Martin J. Kelly, Anne M. Etgen

**Affiliations:** 1 Dominick P. Purpura Department of Neuroscience, Albert Einstein College of Medicine, Bronx, New York, United States of America; 2 Department of Obstetrics & Gynecology and Women's Health, Albert Einstein College of Medicine, Bronx, New York, United States of America; 3 Department of Physiology and Pharmacology, Oregon Health & Science University, Portland, Oregon; University of Nebraska Medical Center, United States of America

## Abstract

**Background:**

Pretreatment with 17β-estradiol (E2) is profoundly neuroprotective in young animals subjected to focal and global ischemia. However, whether E2 retains its neuroprotective efficacy in aging animals, especially when administered after brain insult, is largely unknown.

**Methodology/Principal Findings:**

We examined the neuroprotective effects of E2 and two agonists that bind to non-classical estrogen receptors, G1 and STX, when administered after ischemia in middle-aged rats after prolonged ovarian hormone withdrawal. Eight weeks after ovariectomy, middle-aged female rats underwent 10 minutes of global ischemia by four vessel occlusion. Immediately after reperfusion, animals received a single infusion of either E2 (2.25 µg), G1 (50 µg) or STX (50 µg) into the lateral ventricle (ICV) or a single systemic injection of E2 (100 µg/kg). Surviving pyramidal neurons in the hippocampal CA1 were quantified 1 week later. E2 and both agonists that target non-classical estrogen receptors (G1 and STX) administered ICV at the time of reperfusion provided significant levels of neuroprotection, with 55–60% of CA1 neurons surviving vs 15% survival in controls. A single systemic injection of a pharmacological dose of E2 also rescued approximately 50% of CA1 pyramidal neurons destined to die. To determine if E2 and G1 have similar mechanisms of action in hippocampal neurons, we compared the ability of E2 and G1 to modify CA1 pyramidal neuron responses to excitatory inputs from the Schaffer collaterals recorded in hippocampal slices derived from female rats not subjected to global ischemia. E2 and G1 (10 nM) significantly potentiated pyramidal neuron responses to excitatory inputs when applied to hippocampal slices.

**Conclusions/Significance:**

These findings suggest (1) that middle-aged female rats retain their responsiveness to E2 even after a long period of hormone withdrawal, (2) that non-classical estrogen receptors may mediate the neuroprotective actions of E2 when given after ischemia, and (3) that the neuroprotective efficacy of estrogens may be related to their modulation of synaptic activity in hippocampal slices.

## Introduction

There is growing evidence that natural and synthetic estrogens exert neuroprotective effects in vivo and in vitro [1], [2] suggesting that post-menopausal women might benefit from hormonal supplementation to reduce neurodegeneration associated with brain insults such as stroke or cardiac arrest. However, if synthetic estrogenic compounds designed to treat menopausal symptoms mimic the pleiotropic effects of the natural estrogen 17β-estradiol (E2), the benefits of such hormonal therapy might be outweighed by the reported negative effects of long-term postmenopausal hormone therapy, including higher risk of thrombosis and breast cancer [3], [4].

An alternative to natural estrogens or long-term hormone treatment to protect against brain insults in aging patients would be synthetic molecules that are designed to be administered after an ischemic event and that act with greater selectivity than E2. Selective estrogen receptor modulators (SERMs) are natural or synthetic compounds that have tissue- and species-specific effects distinct from those of E2, acting as estrogen receptor (ER) agonists in some tissues and as antagonists in others [5]. Some synthetic and natural SERMs, such as tamoxifen, raloxifene, bazedoxifene and genistein, provide neuroprotection in vitro and in vivo (for review, see [2]) whereas other SERMs such as LY362321 do not [6]. The phytoestrogen genistein attenuates oxidative stress and neuronal damage following transient global cerebral ischemia in rat hippocampus [7], and tamoxifen protects hippocampal neurons in an oxygen/glucose deprivation model of ischemia in brain slices [8]. Tamoxifen affords neuroprotection through its anti-oxidant properties without interacting directly with ERs [9] or by activating ER-α to reduce glutamate excitotoxicity [8].

STX, a diphenylacrylamide compound (SERM) that does not bind ER-α or ER-β, was originally shown to mimic E2 modulation of hypothalamic ion channels and phospholipase C through the activation of a G-protein coupled receptor (for review see [10]). Another category of estrogenic compounds that do not bind ER-α or ER-β are synthetic molecules that act as agonists at GPR30 ([11], for review see [12]). GPR30 was recently identified as a G protein coupled receptor that binds E2 with high affinity [13]. GPR30 is widely expressed in the brain including the hippocampus [14], and a recent study with GPR30 knockout mice shows that GPR30 is not involved in E2 regulation of female reproductive functions known to be mediated by ERα [15]. Such “non-feminizing estrogens” might become potential candidates for neuroprotection even in male patients if they attenuate neuronal death when given after an ischemic insult.

**Figure 1:**
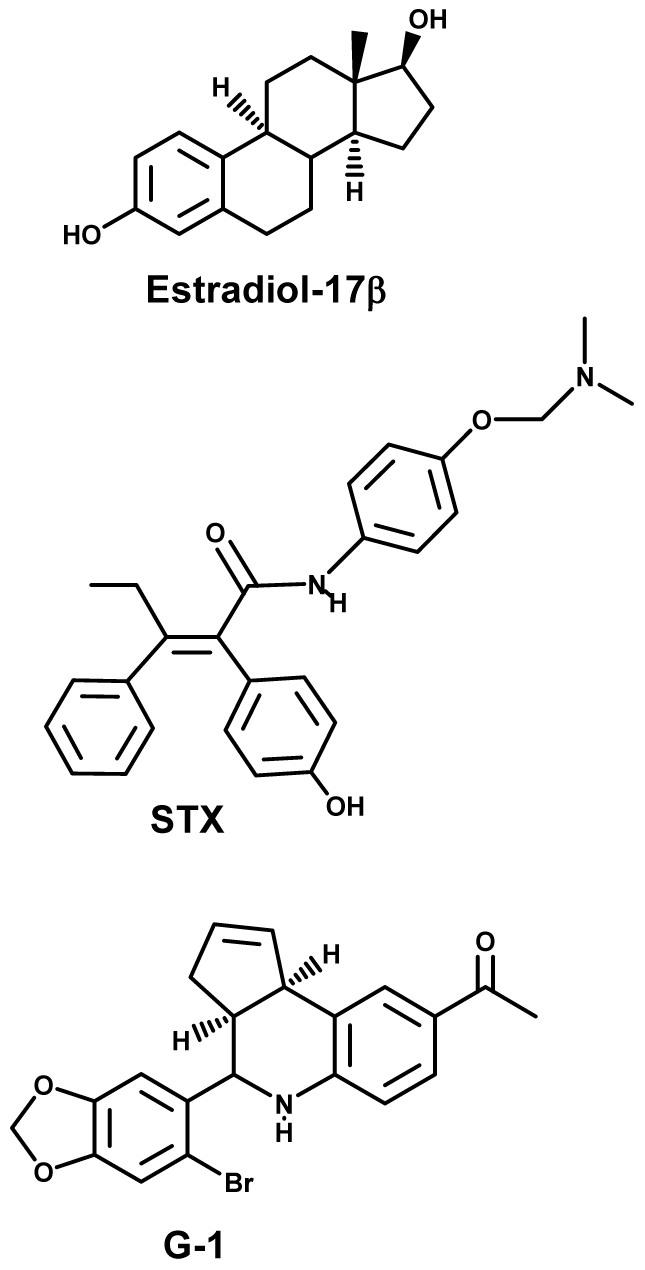
Chemical structure of the natural estrogen 17β-estradiol and non-classical estrogen receptor agonists. G1: GPR30 agonist; STX: diphenylacrylamide compound (SERM) that does not bind ER-α or ER-β.

The present study evaluated the potential neuroprotective actions of the SERM STX [16] and the GPR30 agonist G1 [17] using a well characterized, clinically relevant animal model of transient global ischemia. The demonstration that post-ischemic administration of estrogenic compounds to older animals is neuroprotective would be of great clinical relevance; however, research on neuroprotective effects of natural and synthetic estrogens predominantly use models of global and focal ischemia in young animals combined with longer term (days-weeks) hormone pretreatment (for review see [18]). Therefore, we evaluated the potential neuroprotective action of the natural estrogen E2 and two synthetic estrogenic compounds that do not bind ER-α or ER-β, STX and G1 (see Figure 1 for structures of these compounds), when administered to ovariohysterectomized (OVX) young or middle aged-female rats as a single injection immediately after ischemia. Because the effectiveness of estrogen therapy in protecting brain cells from ischemia-induced death may critically depend on the duration of hormone deprivation [19], [20], [21], we also determined the neuroprotective efficacy of these compounds in middle-aged females subjected to ischemia after long-term (8 weeks) hormone withdrawal.

## Results

### E2, the SERM STX and GPR30 agonist G1 provide neuroprotection when administered after global ischemia

**Figure 2:**
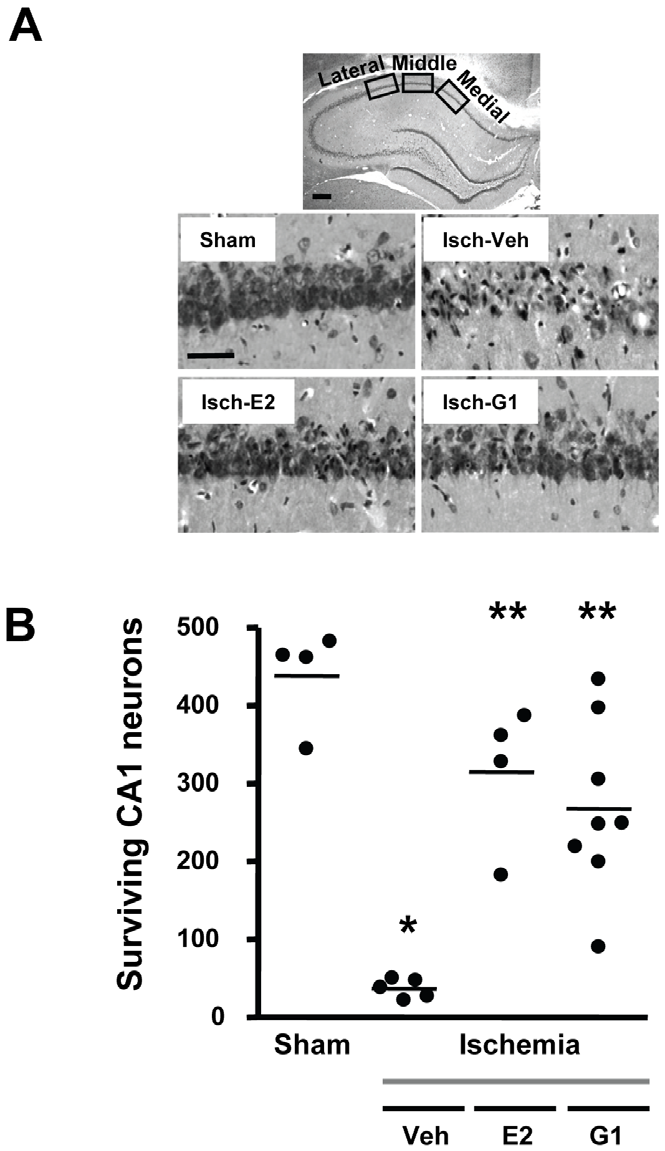
G1 and E2 afford similar levels of neuroprotection when administered to young rats immediately after ischemia. Panel A shows representative photomicrographs of hippocampal neurons in the dorsal CA1 region 7 days after sham surgery or global ischemia (Isch) in young adult female rats that were OVX for 1 week and injected ICV with either E2 (2.25 µg), G1 (50 µg) or vehicle (DMSO) immediately after ischemia. Scale bars: low magnification, 400 µm; higher magnification, 60 µm. Panel B: Quantification of living pyramidal neurons was performed in the CA1 region of the hippocampus 7 days after ischemia. Neurons were counted in 3 sectors (lateral, middle and medial) of the dorsal hippocampus. ANOVA followed by Newman Keuls, * p<0.001 versus sham, ** p<0.001 versus ischemia/vehicle.

The model of global ischemia used in this study produces delayed and highly selective cell death limited to pyramidal neurons in the CA1 subfield of the hippocampus [22]. We previously reported that administration of a high dose (30 µg) of E2 intracerebroventricularly (ICV) to young OVX female rats immediately after global ischemia promoted survival of CA1 hippocampal neurons destined to die and improved cognitive performance [23]. Using the same experimental conditions (young female rats OVX 1 week before ischemia), we now show that a much lower dose of E2 and the selective GPR30 agonist G1, which does not bind to classical ERs [17], confer significant neuroprotection (Figure 2). Counts of surviving CA1 pyramidal neurons were performed 7 days after ischemia on brain sections of sham-operated and ischemic animals injected ICV immediately after ischemia with either E2 (2.25 µg), G1 (50 µg) or vehicle (controls). Ischemic animals treated with vehicle exhibited substantial loss (about 90%) of CA1 pyramidal neurons compared to sham-operated animals (p<0.001). Both E2- and G1-treated ischemic animals had significantly higher numbers of surviving CA1 pyramidal neurons than the ischemic group treated with vehicle (p<0.001). No significant difference in neuronal survival was observed between ischemic rats infused with G1 and E2, suggesting that these two compounds provide the same level of neuroprotection when given acutely just after ischemia.

**Figure 3:**
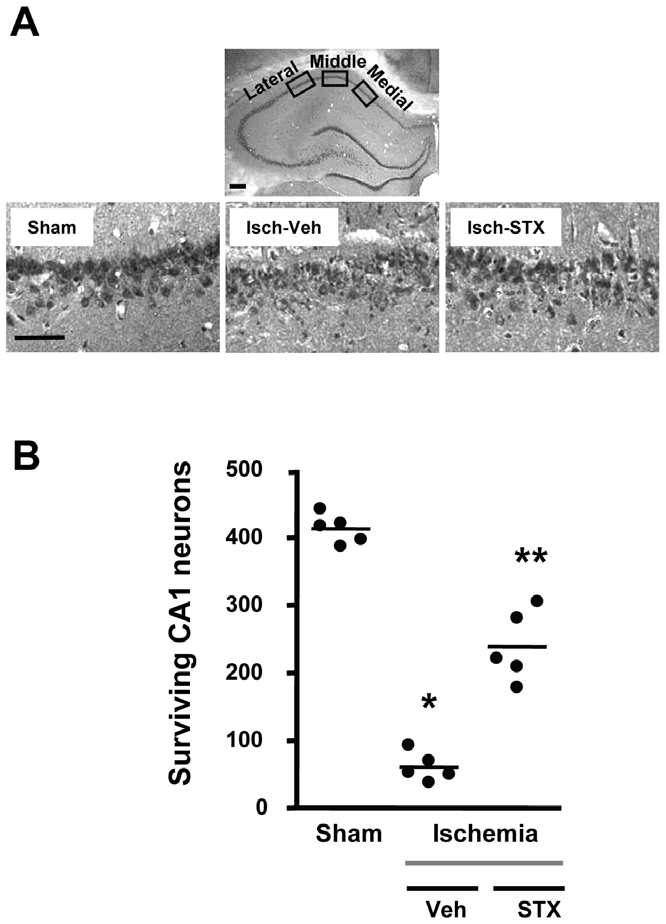
STX affords neuroprotection in short-term OVX, middle-aged rats when administered after ischemia. Middle-aged female rats were subjected to sham surgery or global ischemia (Isch) 1 week after OVX. Panel A shows representative photomicrographs of hippocampal neurons in the dorsal CA1 region 7 days after sham surgery or global ischemia in animals injected ICV immediately after reperfusion with either vehicle (DMSO) or STX (50 µg). Scale bars: low magnification, 400 µm; higher magnification, 60 µm. Panel B: Quantification of living pyramidal neurons was performed in the CA1 region of the hippocampus 7 days after ischemia. Neurons were counted in 3 sectors (lateral, middle and medial) of the dorsal hippocampus. ANOVA followed by Newman Keuls, * p<0.001 versus sham, ** p<0.001 versus ischemia/vehicle.

Determining whether estrogenic compounds administered after ischemia are also neuroprotective in older animals is of high clinical relevance. Indeed, we already showed that chronic supplementation with E2 for 2 weeks before ischemia protects CA1 neurons in middle-aged female rats [24], [25]. Here we show in middle-aged female rats subjected to global ischemia 1 week after OVX that a single ICV injection of the SERM STX (50 µg) immediately after ischemia significantly reduces CA1 pyramidal cell death (Figure 3; p<0.001 vs ischemia plus vehicle). As was the case for E2 and G1 given to young females that were OVX for 1 week, CA1 pyramidal neuron survival in the ischemic group treated with STX remains significantly (p<0.005) lower than in the sham-operated animals.

**Figure 4:**
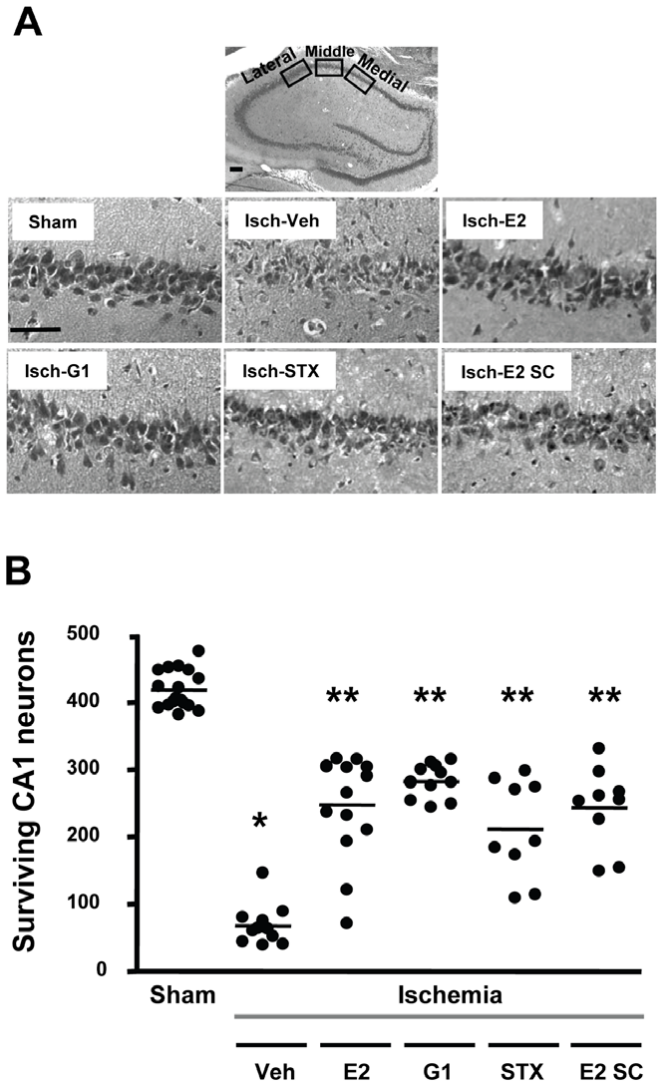
E2, G1 and STX afford neuroprotection in middle-aged females 8 weeks after hormone deprivation. Middle-aged female rats were subjected to sham surgery or global ischemia (Isch) 8 weeks after OVX. Panel A shows representative photomicrographs of hippocampal neurons in the dorsal CA1 region 7 days after sham surgery or global ischemia in animals injected ICV immediately after reperfusion with either vehicle, E2 (2.25 µg), G1 (50 µg), STX (50 µg) or vehicle. Additional animals were injected sc with E2 (100 µg/kg). Scale bars: low magnification, 400 µm; higher magnification, 60 µm. Panel B: Quantification of living pyramidal neurons was performed in the CA1 region of the hippocampus 7 days after ischemia. Neurons were counted in 3 sectors (lateral, middle and medial) of the dorsal hippocampus. ANOVA followed by Newman Keuls, * p<0.001 versus sham, ** p<0.001 versus ischemia/vehicle.

Our recent work with chronic E2 pretreatment in middle-aged female rats showed that histological neuroprotection following global ischemia was maintained even when E2 treatment was delayed for a long time (8 weeks) after OVX [24], [25]. To determine whether acute administration of E2 and synthetic estrogenic compounds such as the GPR30 agonist G1 or the SERM STX are neuroprotective in older animals after prolonged hormonal withdrawal, we tested these compounds in middle-aged females that were OVX for 8 weeks before ischemia (Figure 4). Thus, 8 weeks after OVX, animals were subjected to global ischemia and injected ICV with a single dose of E2 (2.25 µg), G1 (50 µg) or STX (50 µg) immediately upon reperfusion. The number of CA1 pyramidal neurons surviving 7 days later in ischemic animals treated with either E2, G1 or STX was significantly higher (p<0.001) when compared to ischemic animals injected with vehicle. No significant difference (p>0.05) was observed among the three compounds, each of which promoted survival of approximately 50% of CA1 pyramidal neurons. The number of surviving pyramidal cells remained significantly lower (p<0.001) than the number of surviving neurons counted in the CA1 of sham-operated animals. Taken together, these results suggest 1) that acute post-ischemic administration of E2, G1 and STX promotes significant and comparable levels of neuroprotection in a clinically relevant model of global ischemia, and 2) that neuroprotection is maintained even after long-term hormone deprivation.

Because it is not likely that E2 or other compounds can be administered ICV after an ischemic insult in humans, we also determined whether systemic (subcutaneous) administration of a pharmacological E2 dose is neuroprotective in middle-aged females that were OVX for 8 weeks before experimentation.(Figure 4). The number of surviving CA1 pyramidal neurons in animals treated with a single dose of E2 (100 µg/kg) was significantly higher than in ischemic animals treated with vehicle (p<0.001) and did not differ significantly from the live cell counts observed for ischemic animals receiving ICV administration of either E2, G1 or STX.

### The GPR30 agonist G1 mimics short latency E2 potentiation of hippocampal CA1 neuron excitability

**Figure 5:**
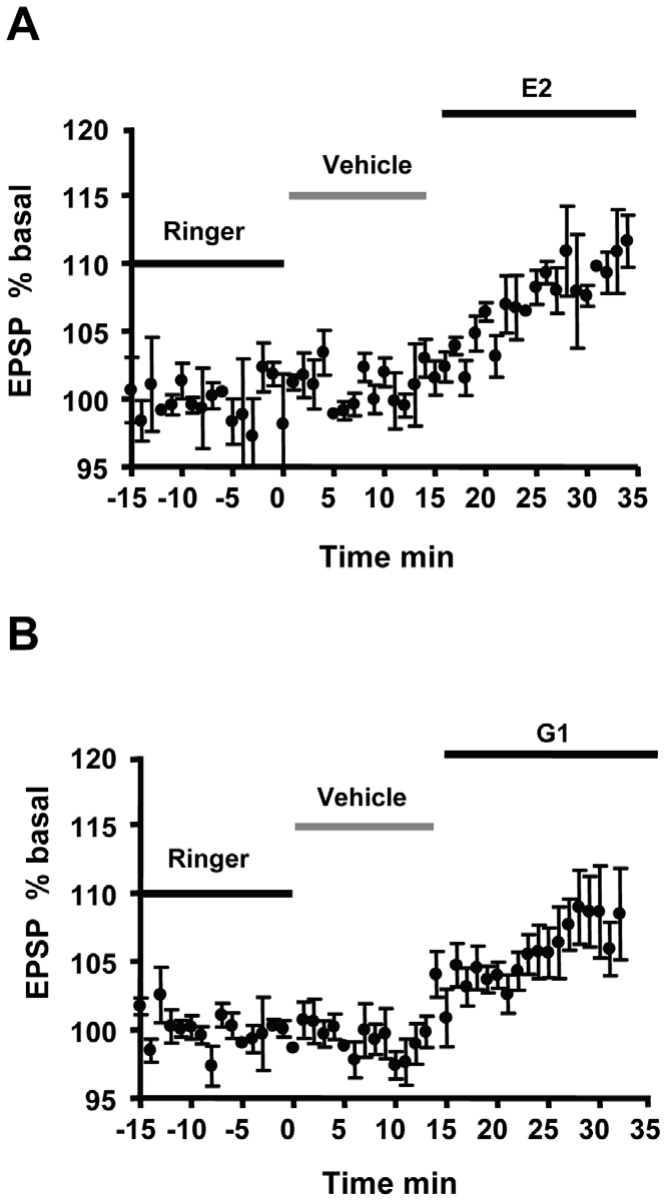
The GPR30 agonist G1 mimics short latency E2 potentiation of hippocampal CA1 neuronal excitability. Changes in field EPSPs in response to E2 or G1 were measured on hippocampal slices prepared from OVX young adult female rats. Schaffer collaterals were activated by monopolar stimulation. Slices were perfused with Ringer's solution until a stable baseline response was obtained. They were then perfused for 15 min with Ringer's solution containing the vehicle and then with 10 nM E2 (A) or G1 (B). Bins of 1 min were plotted. It was frequently possible to record synaptic activity from more than one slice from the same animal in a recording session. Therefore, to test G1, we recorded from 14 slices originating from 9 different OVX females; of these, 9 exhibited an increase in the fEPSP. Similarly, 8 out of 9 slices from 6 different animals responded to E2. Only responding slices are illustrated in the figures.

As a first step in determining whether E2 and the synthetic estrogens have similar actions on hippocampal neurons, we measured the field excitatory postsynaptic potential (fEPSP) generated in the CA1 pyramidal cell layer by Schaffer collateral stimulation in hippocampal slices from young OVX female rats. These animals did not undergo global ischemia. Perfusion of either G1 or E2 (10 nM) significantly enhanced the response of CA1 pyramidal neurons to Schaffer collateral stimulation with a similar latency and magnitude (Figure 5). Two way ANOVA with repeated measures showed a significant effect of time (F = 9.49; p<0.001), a nearly significant interaction between time and drug (F = 1.49; p = 0.05), and no significant difference between the effects of E2 and G1 (F = 0.56; p = 0.65). Both E2 and G1 significantly increased fEPSPs (maximal increase 20% above baseline) with a latency of 7 min, and the facilitation of fEPSP amplitude was maintained throughout the perfusion period. Thus, E2 and G1 exert similar, short latency effects on neuronal excitability. Among 14 slices from OVX females tested with G1, 9 exhibited an increase in the fEPSP. Similarly, 8 out of 9 slices responded to E2. All together, these results are in accord with previous studies which reported that 60-70% of tested hippocampal slices respond to E2 with a modest but significant increase in fEPSP amplitude observed in the first 10 min after hormone application [26], [27], [28].

## Discussion

We report in the present study that central injection of the SERM STX and the GPR30 agonist G1 provides similar levels of neuroprotection as the natural estrogen E2 when administered immediately after global ischemia to middle-aged female rats that had been OVX for 8 weeks before experimentation. Thus, (1) natural and synthetic estrogens are neuroprotective when administered after ischemia, (2) these neuroprotective properties are retained even after long-term hormone deprivation in aging females, and 3) synthetic estrogens that do not bind classical ERs are as neuroprotective as E2. These results are of high clinical relevance because they open up the possibility that such non-feminizing estrogens can be used to treat both male and female patients after brain injury occurs. Because these compounds do not interact with classical ERs, it is also likely that they will not trigger the unwanted side effects of chronic hormone therapy in postmenopausal women, such as increased risk of thrombosis, stroke, cardiovascular events and breast cancer, associated with the activation of classical ERs [3], [4].

It is well established that E2 pretreatment for days to weeks before brain insult is neuroprotective in several preclinical models of focal and global ischemia irrespective of sex, age and method of hormone administration (for review see [18]). There are fewer reports on the ability of E2 to attenuate neuronal death when given after ischemia, and even fewer studies in older animals. Systemic administration of E2 and Premarin (a conjugated equine estrogen preparation) provided neuroprotection when administered after transient or permanent middle cerebral artery occlusion (MCAO) in young male and OVX female rats [29], [30]. Simpkins and colleagues also showed that subcutaneous administration of E2 (100 µg/kg) to OVX young adult female rats 30 min after the onset of occlusion in a model of permanent MCAO significantly reduced lesion volume measured 48 h after ischemia [31]. In the present study, we observe that the same high dose of E2 (100 µg/kg) injected subcutaneously in middle-aged rats immediately after global ischemia rescued a substantial number of hippocampal CA1 pyramidal neurons destined to die at 7 days post-ischemia. Although this dose of E2 would be considered pharmacological, similar high doses of E2 are used clinically to reduce excessive uterine bleeding and to prepare women for embryo transfer during *in vitro* fertilization protocols. Moreover, high doses of estrogens, in combination with a progestin, are given orally for several days to human subjects for post-coital contraception. It is also notable that higher doses of E2 can extend the therapeutic window to 6 hr after focal cerebral ischemia [31]. Therefore, it will be important to establish the therapeutic window and dose requirements for administration of estrogenic compounds in our model of global ischemia.

Among the 66 studies reviewed recently by Strom and colleagues on the neuroprotective effects of E2 in animal models of ischemia, most involved young animals in which the length of time between OVX and hormone administration was no longer than 2 weeks [18]. As stroke and cardiac arrest occur more frequently in older individuals [32], it is clinically relevant to study E2 neuroprotection in the aging brain. Moreover, because the risk of stroke and cardiovascular events increases with time after menopause in women [32], it is also important to determine whether estrogenic compounds still afford neuroprotection after long-term ovarian hormone deprivation. Several studies confirmed that E2 reduces the extent of neuronal damage after focal or global ischemia when administered to middle-aged or reproductively senescent female rats at short intervals after OVX [24], [33], [34], [35]. However, some data from clinical and preclinical research support the hypothesis that there may be a narrow time frame for E2 to retain its beneficial actions following ovarian hormone withdrawal [19], [20], [21]. For example, E2 supplementation before ischemia was no longer efficacious in protecting the brain against permanent MCAO in young rats if they were OVX for 10 weeks before insult [21]. In contrast, in our model of transient global ischemia, E2 pretreatment of middle-aged females for 2 weeks starting 8 weeks after OVX rescued the same number of hippocampal CA1 neurons as in females receiving E2 beginning immediately or 1 week after OVX [24], [25]. In agreement with our earlier results, the present study shows that central or systemic E2 administration affords robust neuroprotection in a model of global ischemia when administered as a single dose immediately after reperfusion even after 8 weeks of hormone deprivation.

As was the case with long-term E2, we found that STX provides comparable neuroprotection in middle-aged female rats at either 1 week or 8 weeks following OVX (compare Figures 3 and 4). G1, which was neuroprotective when administered to young adults 1 week after OVX (Figure 2), also rescued neurons in middle-aged animals after 8 weeks of hormone withdrawal (Figure 4). Thus, whether there is a narrow time frame for E2 to retain its beneficial actions following ovarian hormone withdrawal might depend on the type of brain insult and/or the brain region affected. Our results suggest that the duration of hormone withdrawal does not reduce the benefits of post-ischemic estrogen administration in a rat model of global ischemia when high doses of E2 are used. The importance of using higher E2 doses is suggested by a recent report that long-term pretreatment with very low levels of E2 (to mimic diestrus) failed to reduce CA1 pyramidal cell death induced by global ischemia in middle-aged female rats that were OVX for 10 weeks before insult [20].

Another important outcome of this study is the finding that estrogenic compounds that do not bind classical ERs and that potentially activate G-proteins can afford neuroprotection equivalent to natural E2. Similar to studies on the neuroprotective effect of other non-feminizing estrogens in focal ischemia [36], the results reported here may have a significant impact on therapeutic approaches. First, because E2 also reduces the extent of neuronal damage in males subjected to ischemic injury [37], [38], [39], [40], it may be possible to develop neuroprotective estrogen analogs that could be used in male patients without the risk of feminization mediated by the activation of the classical nuclear receptors. Second, activation of the classical nuclear receptors is known to increase the risk of hormone dependent breast cancer [41]. Thus, understanding the cellular and molecular mechanisms by which synthetic estrogens that do not bind to classical ERs afford neuroprotection is a critical area for future research.

STX is a synthetic SERM (diphenylacrylamide compound) that was shown to mimic, in the hypothalamus, the effects of E2 mediated by the activation of an unidentified G-protein coupled receptor [10], [16]. STX does not bind to or activate the classical nuclear ERs [42], [43] and was recently shown to activate the MAPK and PI3K pathways in endometrial cells [42]. G1 is a specific GPR30 agonist that does not bind to the classical nuclear receptors [17] and that also activates the MAPK [44], [45] and PI3K signaling pathways [17]. Thus, one might hypothesize that STX and G1 promote neuroprotection through the activation of the MAPK and/or PI3K cell signaling pathways, both of which are known to be activated by E2 and to promote neuroprotection following global ischemia [46], [47]. Interestingly, SERMs such as genistein and hydroxytamoxifen, which are potential neuroprotective agents [2], also bind to GPR30 [13]. Because STX modulation of inwardly rectifying potassium currents in hypothalamic neurons is not impaired in GPR30 null mice [10], G1 and STX might exert their neuroprotective effects through the activation of two distinct G protein-coupled receptors, the one binding STX remaining to be identified. Linking the neuroprotective actions of such estrogenic compounds to activation of G protein-coupled receptors in the brain is highly novel and might lead to the development of a new class of neuroprotective agents.

Activation of G protein coupled receptors can modulate neuronal excitability. Indeed, we previously reported that the GPR30 agonist G1 mimics the short latency E2 facilitation of synaptic transmission, as measured by changes in whole cell EPSPs, in CA1 pyramidal neurons in hippocampal slices [48]. Presently, we found that G1 also mimics E2-dependent increases in fEPSPs, which corroborates the hypothesis that GPR30 is present and functional in pyramidal neurons of the rat hippocampus and that this receptor may mediate some of the short latency actions of E2 on neuronal excitability. Whether the effects of G1 and E2 on synaptic efficacy are related to their neuroprotective effects remains to be examined. Enhanced neuronal excitability might be predicted to exacerbate ischemic injury. However, the possibility that neuroprotective effects of estrogenic compounds could involve a glutamatergic excitatory pathway is suggested by the observations that preconditioning with sub-toxic doses of NMDA promotes neuroprotection in vitro by enhancing hippocampal neuronal excitability [49]. Moreover, several studies suggest that NMDA receptor activation is causally related to ischemic preconditioning in vivo ([50], for review see [51]).

In conclusion, we report here for the first time that synthetic estrogenic compounds that do not bind the classical ER-α and ER-β afford robust neuroprotection when administered immediately after global ischemia. This protection, like that obtained with E2, is still observed in middle-aged female rats even after a prolonged period of hormone deprivation. This should encourage the search for therapeutic agents that are safe and neuroprotective in both female and male patients when administered after brain insult and that do not trigger the unwanted effects of estrogens mediated by the activation of classical ER-α and ER-β.

## Materials and Methods

### Animals

Young (180–200 g, ∼2 months) or middle-aged (retired breeders, 9–11 months) Sprague Dawley female rats were purchased from Charles River (Wilmington, MA) and housed 2–4 per cage on a 14/10 h light/dark cycle with ad lib access to food and water. All procedures involving animals were performed in accordance with NIH guidelines and were approved by the Institutional Animal Care and Use Committee of the Albert Einstein College of Medicine.

### Global ischemia and hormone treatment

One or 8 weeks after ovariohysterectomy (OVX), animals were subjected to transient global ischemia by 4 vessel occlusion [22]. Briefly, the vertebral arteries were exposed through a midline occipital–suboccipital incision and coagulated with bipolar cauterization between the first and second cervical vertebral bodies. This procedure by itself has no effect on cerebral blood flow but prevents collateral circulation to the forebrain during subsequent transient carotid artery occlusion. The same day, a 3-0 silk thread was also looped around the carotid arteries to facilitate subsequent occlusion. Twenty-four hours later, the neck wound was reopened, and transient global ischemia was induced by temporary, bilateral occlusion of the common carotid arteries for 10 min followed by reperfusion. Animals were lightly anesthetized to place the microarterial clamps but were awake and spontaneously ventilating during the 10 min of occlusion. Ischemia was ensured by monitoring the loss of righting reflex and bilateral pupil dilation of each subject during carotid occlusion. Sham-operated rats had their vertebral arteries coagulated and underwent all other surgical procedures except for carotid artery occlusion. All surgical procedures were performed under isofluorane (4% induction, 2% maintenance in 70% N_2_O:30% O_2_). A rectal probe was inserted to maintain stable core body temperature (36.5–37.5°C) using a heating pad.

Drugs were administered immediately upon reperfusion by either ICV or subcutaneous injection. For ICV injections, under isofluorane anesthesia, the needle (26 g) of a Hamilton syringe was lowered into the right lateral ventricle using coordinates (AP:−0.8, M/L:−1.5, D/V:−3.6) from Paxinos [52] and Bregma as a landmark. A total of 5 µl were injected at a rate of 1 µl/min, and then the needle was left in place for 1 additional min. Animals were injected ICV with either β-cyclodextrin encapsulated E2 (Sigma) dissolved in sterile saline (corresponding to 2.25 µg of free E2), STX (50 µg), G1 (Calbiochem, 50 µg), or the appropriate vehicles (β-cyclodextrin in saline for E2 or 100% DMSO for G1 and STX). Stock solutions of G1 and STX at 10 mg/ml in 100% DMSO were kept at −20°C until the day of injection. For subcutaneous injection, E2 was solubilized in 100% ethanol at 10 mg/ml, then further diluted in peanut oil and shaken overnight at 37°C to evaporate the ethanol. A final dose of 100 µg/kg of E2 was injected.

### Quantification of surviving hippocampal CA1 pyramidal neurons

Seven days after global ischemia, rats were transcardially perfused using 0.9% saline with heparin followed by ice cold 10% phosphate buffered formalin (Fisher Scientific, Pittsburgh, PA). Brains were removed, placed in formalin at 4°C overnight, fixed in 30% sucrose in phosphate buffered saline at 4°C for 48 h and then frozen at −80°C. Coronal sections (20 µm) were cut at the level of the dorsal hippocampus (3.3–4.0 mm posterior from bregma), and 4 sections per animal at 140 µm intervals were mounted and stained with hematoxylin and eosin. The dorsal hippocampus is more vulnerable to ischemic damage than the ventral hippocampus [53]; hence, counts of surviving neurons were performed only at the dorsal level. Medial, middle, and lateral sectors from the CA1 region of the left and right hippocampus were photographed at 40X magnification using a Nikon microscope and digital camera. As previously described [24] and shown in Fig. 2A, a microscope counting grid (250 µm×250 µm) was positioned a few cells medial from CA2 neurons (lateral sector), at the apex of the CA1 (middle sector) and on the upswing of CA1 in an area clearly distinct from subiculum (medial sector). Digital images were opened in Adobe Photoshop, and the number of viable pyramidal neurons in these regions of interest was counted. Viable neurons (not eosinophilic) had rounded cell bodies and clearly visible nucleoli. Pyknotic and shrunken neurons were not counted. Counts of surviving neurons were summated over right and left hemispheres. All cell counts were carried out by an investigator who was blind to the animals' treatment.

### Electrophysiology

Female rats (180–200 g) that had been OVX for 5–10 days were anesthetized with isofluorane, and the brains rapidly removed into chilled cutting solution consisting of (in mM) 215 sucrose, 2.5 KCl, 20 glucose, 26 NaHCO_3_, 1.6 NaH_2_PO_4_, 1 CaCl_2_, 4 MgCl_2_, and 4 MgSO_4_. Hippocampi were dissected and cut into 400 µm transverse sections on a DTK-2000 vibrating microslicer (Dosaka EM Co., Ltd., Japan) using standard methods. Slices from the dorsal hippocampus were submerged in a holding chamber, and the sucrose cutting solution was gradually shifted to recording solution pre-warmed to 25°C (ACSF in mM: 124 NaCl, 2.5 KCl, 10 glucose, 26 NaHCO_3_, 1 NaH_2_PO_4_, 2.5 CaCl_2_, and 1.3 MgSO_4_, pH 7.4) over 15–20 min in the incubation chamber. At least 60 min were allowed for recovery before recording. All solutions were gassed with 95% O_2_/5% CO_2_. All recordings were conducted at 25°C in a submersion-type recording chamber (0.8 ml volume) perfused at 2.4 ml/min (PH 3 recording chamber, SH-27B inline heater, TC-344B temperature controller, Warner Instruments, LLC, Hamden, CT). Slices were selected if they exhibited stable fEPSPs in response to stimulation of the Schaffer collaterals. It was possible to record from more than one slice from the same animal during a single recording session.

Recording of extracellular field potentials evoked in CA1 after electrical stimulation (0.05 Hz) of the Schaffer collateral pathway was performed with a monopolar stimulating electrode filled with extracellular ACSF and a glass recording electrode filled with 1 M NaCl, both positioned in stratum radiatum and visualized using an Eclipse E600FN microscope (Nikon, Melville, NY). The recording and stimulating electrodes were placed at the same depth (60 µm) in the slice, and the distance between them was kept constant (180 µm). Recordings were performed with a MultiClamp 700A amplifier (Molecular device, Sunnyvale, CA), and output signals were filtered at 3 kHz. Data were digitized (20 kHz sampling frequency) and analyzed online using a macro written in IgorPro (Wavemetrics, Portland, OR). Field excitatory postsynaptic potential (fEPSP) initial slopes (mV/ms) were measured and expressed as percent change from baseline values. After at least 20 min of stable recording, the recording solution was switched to recording solution containing 0.01% DMSO; 15 min later hippocampal slices were perfused with recording solution containing 0.01% DMSO and either E2 or G1 at 10 nM. After every experiment, the inline heater, tubing and recording chamber were washed thoroughly with ethanol and copious water.
